# COVID-19 Infodemic and Impacts on the Mental Health of Older People: Cross-sectional Multicenter Survey Study

**DOI:** 10.2196/42707

**Published:** 2023-05-17

**Authors:** Patricia Rodrigues Braz, Tiago Ricardo Moreira, Andréia Queiroz Ribeiro, Luciane Ribeiro de Faria, Fabio da Costa Carbogim, Vilanice Alves de Araújo Püschel, Jack Roberto Silva Fhon, Eduarda Rezende Freitas, Ione Carvalho Pinto, Fabiana Costa Machado Zacharias, Gylce Eloisa Cabreira Panitz Cruz, Richardson Miranda Machado, Rosimere Ferreira Santana, Priscilla Alfradique de Souza, Graziele Ribeiro Bitencourt, Alexandre Favero Bulgarelli, Ricardo Bezerra Cavalcante

**Affiliations:** 1 Universidade Federal de Juiz de Fora Juiz de Fora Brazil; 2 Universidade Federal de Viçosa Viçosa Brazil; 3 Universidade Federal de Juiz de Fora Juiz de Fora - MG Brazil; 4 Universidade de São Paulo São Paulo Brazil; 5 Universidade Católica de Brasília Brasília Brazil; 6 Universidade de São Paulo Ribeirão Preto Brazil; 7 Universidade Federal de São João Del Rei Divinópolis Brazil; 8 Universidade Federal Fluminense Niterói Brazil; 9 Universidade Federal do Estado do Rio de Janeiro Rio de Janeiro Brazil; 10 Universidade Federal do Rio de Janeiro Macaé Brazil; 11 Universidade Federal do Rio Grande do Sul Porto Alegre Brazil

**Keywords:** information dissemination, health communication, COVID-19, COVID-19 pandemic, public health, health of older people, mental health

## Abstract

**Background:**

The COVID-19 pandemic received widespread media coverage due to its novelty, an early lack of data, and the rapid rise in deaths and cases. This excessive coverage created a secondary “infodemic” that was considered to be a serious public and mental health problem by the World Health Organization and the international scientific community. The infodemic particularly affected older individuals, specifically those who are vulnerable to misinformation due to political positions, low interpretive and critical analysis capacity, and limited technical-scientific knowledge. Thus, it is important to understand older people’s reaction to COVID-19 information disseminated by the media and the effect on their lives and mental health.

**Objective:**

We aimed to describe the profile of exposure to COVID-19 information among older Brazilian individuals and the impact on their mental health, perceived stress, and the presence of generalized anxiety disorder (GAD).

**Methods:**

This cross-sectional, exploratory study surveyed 3307 older Brazilians via the web, social networks, and email between July 2020 and March 2021. Descriptive analysis and bivariate analysis were performed to estimate associations of interest.

**Results:**

Major proportions of the 3307 participants were aged 60 to 64 years (n=1285, 38.9%), female (n=2250, 68.4%), and married (n=1835, 55.5%) and self-identified as White (n=2364, 71.5%). Only 295 (8.9%) had never started or completed a basic education. COVID-19 information was mainly accessed on television (n=2680, 81.1%) and social networks (n=1943, 58.8%). Television exposure was ≥3 hours in 1301 (39.3%) participants, social network use was 2 to 5 hours in 1084 (32.8%) participants, and radio exposure was ≥1 hour in 1223 (37%) participants. Frequency of exposure to social networks was significantly associated with perceived stress (*P*=.04) and GAD (*P*=.01). A Bonferroni post hoc test revealed significantly different perceived stress in participants who were exposed to social networks for 1 hour (*P*=.04) and those who had no exposure (*P*=.04). A crude linear regression showed that “some” social media use (*P*=.02) and 1 hour of exposure to social media (*P*<.001) were associated with perceived stress. Adjusting for sociodemographic variables revealed no associations with this outcome variable. In a crude logistic regression, some social media use (*P*<.001) and 2 to 5 hours of exposure to social media (*P*=.03) were associated with GAD. Adjusting for the indicated variables showed that some social network use (*P*<.001) and 1 hour (*P*=.04) and 2 to 5 hours (*P*=.03) of exposure to social media were associated with GAD.

**Conclusions:**

Older people, especially women, were often exposed to COVID-19–related information through television and social networks; this affected their mental health, specifically GAD and stress. Thus, the impact of the infodemic should be considered during anamnesis for older people, so that they can share their feelings about it and receive appropriate psychosocial care.

## Introduction

The COVID-19 pandemic has been described as the greatest health crisis of the 21st century and is recognized as a public health emergency of international importance [1,2]. COVID-19 is a highly contagious infectious disease. Its main symptoms are coughing, sore throat, runny nose and nasal congestion, fatigue, anosmia, ageusia, diarrhea, headache, and skin rashes. Other symptoms are more severe, such as severe acute respiratory syndrome, shock, and multiple organ dysfunction. In Brazil, the disease caused more than 700,000 deaths [3].

Due to the novelty of the diagnosis, the initial lack of information, and the rapid dissemination of the disease worldwide, as well as the number of deaths and symptomatic cases, the COVID-19 pandemic quickly received wide media coverage. Excessive information related to the disease caused problems secondary to the pandemic, constituting an “infodemic” that impacted the lives of individuals. The World Health Organization and the scientific communities of different countries consider infodemics a serious public health problem [4].

Infodemics are defined by the volume of information associated with a specific subject multiplying quickly in a short time [4]. In this context, Wardle and Derakshan [5] described different terms related to the disclosure of information, constituting what the authors call “an ecosystem of disinformation.”

This ecosystem is composed of 3 large groups: “misinformation,” “malinformation,” and “disinformation.” Misinformation refers to false information disseminated without the primary intention of causing harm. Malinformation is based on legitimate content but is used to cause harm; information is taken out of context or manipulated to achieve the goal of causing harm [5]. By contrast, disinformation is the creation of false information designed specifically for a harmful purpose [5,6].

In Brazil, the political context added to the COVID-19 infodemic and led to the emergence of conspiracy theories and misinterpretations of data and scientific research that were grounded in denialism, which requires the acceptance of interventions without scientific validation. Denialist perspectives on COVID-19 are at the heart of antiscientific and even antihistorical thinking, with the rhetoric of the antivaccine movement being an example. This discourse is characterized by being cyclical and permeated by ethical-political and ideological issues, in which actions are intentional [7,8].

Among all age groups, the infodemic has had the greatest impact on the lives of older individuals. In addition to their political positions and behavior, older people who have low interpretive and critical analysis capacity and limited technical-scientific knowledge tend to generate more misinformation and be exposed to more misinformation, which can impact their mental health. Older individuals represent the largest proportion of functionally illiterate individuals in Brazil, and the illiteracy rate in people aged 60 years or older reaches 10.3% among White people, increasing to 27.5% among other racial groups; moreover, 53% of people aged between 50 and 64 years are considered functionally illiterate, which makes them vulnerable to being targets of misinformation, as well as being its main propagators [9].

Older people are not digital natives but are increasingly active and, consequently, are gradually starting to use computerized media and gain access to other media. Internet use has grown most rapidly among older people over the years, increasing by 56% between 2015 and 2017 [10] and by 97% by 2021 [11].

Given the above, it is important to understand how older people react to information about COVID-19 disseminated by the media, what effects this information has on their lives, and how it can affect their mental health. Thus, we conducted this study, which is derived from an international multicenter study titled “COVID-19 infodemic and its repercussions on the mental health of the elderly during and postpandemic: A multicenter study Brazil/Chile/Peru/Colombia/Mexico and Portugal.” We aimed to describe the profile of exposure to information on COVID-19 among older Brazilian individuals and determine the mental health repercussions of this exposure by screening for perceived stress and generalized anxiety disorder (GAD). The following hypothesis guided the collection and analysis of quantitative data: There are associations between variables related to the COVID-19 infodemic, GAD, and perceived stress among older Brazilian people.

With regard to screening for perceived stress and GAD, it is necessary to understand aspects of these conditions that are accentuated in the context of an infodemic and pandemic.

Stress has multiple etiologies and can be understood as the result of exposure to events considered stressful that exceed the body’s ability to control or manage them; this generates important behavioral symptoms, such as fear, avoidance behaviors, irritability, and repetitive nightmares. Stress is also a symptom of several mental disorders [12].

The diagnosis of GAD is based on excessive anxiety, worry, and apprehensive anticipation that occur on most days for at least 6 months and are related to a variety of events or activities that the individual finds it difficult to control. Anxiety and worry are generally associated with signs and symptoms of restlessness, fatigability, difficulty concentrating, irritability, muscle tension, and sleep disturbance; these signs and symptoms persist and cause clinically significant distress or impairment in social and professional functioning [12].

## Methods

### Overview

This paper is derived from an international multicenter study that is still ongoing, titled “COVID-19 infodemic and its repercussions on the mental health of the elderly during and postpandemic: A multicenter study Brazil/Chile/Peru/Colombia/Mexico and Portugal,” which seeks to analyze the COVID-19 infodemic and its repercussions on the mental health of older individuals.

This is a cross-sectional, quantitative, exploratory study conducted among people aged 60 years or older in several Brazilian municipalities. Participants eligible for this study were older Brazilians who had access to the internet and social networks.

The data collection instruments were delivered through a web-based survey between July 2020 and March 2021. The access link was sent by email and through social networks; up to 3 attempts were made over 3 months. Mediation with older people was performed in collaboration with scientific societies for geriatrics and gerontology, health care units, and associations of retirees; direct contact was also made with older people who were already being monitored through research and outreach activities at the collaborating research centers.

When they first accessed the link, the participants were directed to a digital informed consent form where they either accepted or refused to participate in the study. The choice to participate or not participate in the study was automatically recorded in a database generated by the web-based survey. Those who chose to continue participating in the study gained access to the research questions.

The data collection instrument was adapted from studies by Ahmad and Murad [13] and Gao et al [14] and measured demographic and socioeconomic variables. In addition, it included variables related to the COVID-19 infodemic that measured types of media accessed, including social networks, radio, and television, and the time of exposure to these media as frequency and hours. The Perceived Stress Scale (PSS) was used to screen for perceived stress and was analyzed as a continuous variable [15]. The Geriatric Anxiety Inventory (GAI) was used to screen for GAD and was analyzed according to studies validated and conducted in Brazil; a cutoff score of 13 indicated the presence of GAD [16].

The final version of the database was transferred from Microsoft Excel (Microsoft Corp) to SPSS (version 23.0; IBM Corp). The treatment and descriptive analysis procedures were performed through sociodemographic characterization of the participants and with reference to variables related to exposure to COVID-19–related news and information in the media. For the qualitative variables, the absolute and relative frequencies were estimated. For the quantitative variables, measures of position (mean and median) and dispersion (SD, IQR, and minimum and maximum) were estimated according to data distribution (symmetric or asymmetric).

Responses to the PSS questionnaire were analyzed by calculating position and dispersion measures. The estimated prevalence of GAD among the population was determined according to a cutoff point adopted for the analysis of the GAI.

A 1-way analysis of variance (ANOVA) was used to determine differences in mean perceived stress in the participants based on infodemic exposure and sociodemographic characteristics using the Bonferroni post hoc test and a significance level of *P*<.05. Multiple linear regression analysis was performed to estimate the crude and adjusted regression coefficients with 95% CIs for the association between the independent variables of interest and the perceived stress outcome.

The associations between infodemic variables and outcomes related to the geriatric anxiety screening were assessed using the chi-square test. Subsequently, crude and adjusted multiple logistic regression analyses were performed to identify associations between the independent variables of interest and the GAD outcome with the 95% CI. The significance level adopted in all tests was 5%.

### Ethical Considerations

The study was approved by the Brazilian National Research Ethics Committee (Comissão Nacional de Ética em Pesquisa) on March 7, 2020 (CAAE 31932620.1.1001.5147; opinion 4.134.050). Data collection was initiated after approval.

This study complied with all ethical and legal requirements specific to investigations involving human beings, in line with the regulatory provisions of Brazilian Resolution No 466/12 of the National Health Council. The interviewees were guaranteed anonymity and codenames were used to represent them in the study. The participants were informed about the study’s objectives, its justification, and the research procedures, and it was explained to them that participation was voluntary, without financial advantages or expenses. Findings will be disclosed only at scientific events or in journals.

The data and instruments used in the research will be archived with the responsible researcher for a period of 5 years and will be destroyed after that period. Authorizations were requested from the institutions involved to carry out the research. The consent to said documents relies on the performance of the study, description of the methods, and the dissemination of results exclusively in events or journals of a scientific nature.

## Results

A total of 3307 older individuals participated in the study, of whom 2250 (68.4%) were female, 1285 (38.9%) were aged between 60 and 64 years, 1835 (55.5%) were married, and 2364 (71.5%) were White. Housing data showed that 3160 (95.6%) lived in an urban area and 1886 (57%) lived in homes with 1 or 2 people. Only 295 (8.9%) had not started or completed basic education, while 645 (19.5%) had completed higher education. The majority, 1343 (40.6%), used free and paid health services. In addition, 2437 (73.8%) responded that the pandemic did not affect their monthly income (Table 1).

The most used resources to access news and information about COVID-19 during the day among the 3307 participants were television, used by 2680 (81.1%), and social networks, used by 1943 (58.8%); only 876 (26.5%) used the radio. Participants also self-reported their frequency of exposure to information and news about COVID-19 over a period of 1 week (ie, 7 days), ranging from “no exposure” to “frequent exposure.” Television was reported as a frequent source by 1473 (44.5%) participants, while social networks were reported as sometimes being a source by 1464 (44.3%) patients. In contrast, the radio was not a source of exposure for the majority of participants (n=1956, 59.1%). Television exposure was 3 hours or more for 1301 (39.3%) participants, social network exposure was 2 to 5 hours for 1084 (32.8%) participants, and radio exposure was 1 hour or more for 1223 (37%) participants (Table 2).

An ANOVA testing the association of perceived stress with infodemic variables showed a significant association between perceived stress and frequency of exposure to social networks (*P*=.04). It is also noteworthy that the mean score for perceived stress was higher (mean 20.84, SD 9.55) among those who had no exposure to social networks. The Bonferroni post hoc test revealed that the perceived stress of participants who were exposed to social networks for 1 hour (*P*=.04) and those who had no exposure to social networks (*P*=.04) differed significantly. There was also a significant association with hours of exposure to news and information about COVID-19 on social networks (*P*=.02; Table 3).

Approximately 143 of 822 (17.4%) of participants who were not exposed to social networks and 173 of 1021 (16.9%) who were frequently exposed to social networks had a GAI score that indicated GAD (*P*<.001). A significant association was found between geriatric anxiety and hours of exposure to news and information about COVID-19 on social networks (*P*=.01). Among the participants, 73 of 395 (18.5%) who were frequently exposed to radio as a means of information and 105 of 560 (18.8%) who were exposed to social networks for 6 hours or more had a GAI score that indicated GAD (Table 4).

Logistic regression analyses were performed a GAI score indicating GAD as the outcome variable, and linear regression was used for perceived stress to estimate the raw and adjusted regression coefficients for the predictor variables (ie, the sociodemographic variables: age group, gender, education, cohabitation status, and income changes) and the infodemic variables.

The crude linear regression showed that some frequency of exposure to social networks (*P*=.02) and 1 hour of exposure to social networks (*P*<.001) were associated with perceived stress. In the analysis adjusted for the sociodemographic variables, no associations were found with the outcome variable. In the crude logistic regression, some frequency of exposure to social networks (*P*<.001) and 2 to 5 hours of exposure to social networks (*P*=.03) were also associated with GAD. In the adjusted analysis for the indicated variables, some frequency of exposure to social networks (*P*<.001), 1 hour of exposure to social networks (*P*=.04), and 2 to 5 hours of exposure to social networks (*P*=.03) were associated with a GAI score indicating GAD (Table 5).

**Table 1:** Sociodemographic profile of participants in this study (n=3307).

Variables		Participants, n (%)
**Gender**		
	Female	2250 (68.4)
	Male	1039 (31.6)
	Prefer not to declare	18 (0.5)
**City**		
	Other	544 (16.4)
	Juiz de Fora, Minas Gerais	470 (14.2)
	São Paulo, São Paulo	412 (12.5)
	Porto Alegre, Rio Grande do Sul	397 (12)
	Divinópolis, Minas Gerais	381 (11.5)
	Rio de Janeiro, Rio de Janeiro	352 (10.6)
	Viçosa, Minas Gerais	335 (10.1)
	Ribeirão Preto, São Paulo	251 (7.5)
	Brasília, Distrito Federal	165 (5)
**Age group (years)**		
	60 to 64	1285 (38.9)
	65 to 69	921 (27.9)
	70 to 74	503 (15.2)
	75 to 79	334 (10.1)
	80 or older	264 (8)
**Marital status**		
	Married or living together	1835 (55.5)
	Widowed	598 (18.1)
	Separated	509 (15.4)
	Single	365 (11)
**Race**		
	White	2364 (71.5)
	Non-White	943 (28.5)
**Cohabitation**		
	Living alone	587 (17.8)
	Living with 1 or 2 people	1886 (57)
	Living with 3 or more people	834 (25.2)
**Own residence**		
	Yes	2756 (83.3)
	No	551 (16.7)
**Area of residence**		
	Urban	3160 (95.6)
	Rural	147 (4.4)
**Maximum education**		
	Did not complete basic education	295 (8.9)
	Basic or elementary education	713 (21.6)
	Secondary education	718 (21.7)
	Completed higher education	645 (19.5)
	Specialization	512 (15.5)
	Master’s, doctoral, or postdoctoral degree	424 (12.8)
**Health services used**		
	Only paid health services (including health insurance)	1133 (34.3)
	Both (free and paid)	1343 (40.6)
	Only free health services	814 (24.6)
	None	17 (0.5)
**Receives retirement or pension income**		
	Yes	2565 (77.6)
	No	740 (22.4)
**Pandemic altered income**		
	No	2437 (73.8)
	Yes, my income decreased	787 (23.8)
	Yes, my income increased	80 (2.4)

**Table 2:** Characteristics of the methods most commonly used to access news and information about COVID-19 and the frequency and hours of exposure (n=3307).

Characteristics		Participants, n (%)
**Social network exposure (n=3303)**		
	Yes	1943 (58.8)
	No	1361 (41.2)
**Frequency of exposure**		
	None	822 (24.9)
	Sometimes	1464 (44.3)
	Often	1021 (30.9)
**Hours of exposure to news and information about COVID-19 on social networks**		
	None	848 (25.6)
	1	811 (24.5)
	2 to 5	1084 (32.8)
	6 or more	560 (16.9)
**Television exposure (n=3304)**		
	Yes	2680 (81.1)
	No	624 (18.9)
**Frequency of exposure**		
	None	394 (11.9)
	Sometimes	1440 (43.5)
	Often	1473 (44.5)
**Hours of exposure to news and information about COVID-19 on television**		
	None	431 (13)
	1	884 (26.7)
	2	685 (20.7)
	3 or more	1301 (39.3)
**Radio exposure (n=3304)**		
	Yes	876 (26.5)
	No	2429 (73.5)
**Frequency of exposure**		
	None	1956 (59.1)
	Sometimes	956 (28.9)
	Often	395 (11.9)
**Hours of exposure to news and information about COVID-19 on the radio**		
	None	2083 (63)
	1 or more	1223 (37)

**Table 3:** Differences in mean Perceived Stress Scale score and infodemic variables (n=3307).

Variables			Perceived Stress Scale score, mean (SD)	*P* value^a^	
**Frequency of exposure to social networks**					.04
	None	20.84 (9.55)			
	Sometimes	19.89 (9.27)			
	Often	20.61 (9.82)			
**Frequency of exposure to television**					.33
	None	20.03 (9.51)			
	Sometimes	20.16 (9.38)			
	Often	20.62 (9.65)			
**Frequency of exposure to radio**					.89
	None	20.36 (9.57)			
	Sometimes	20.40 (9.33)			
	Often	20.13 (9.72)			
**Hours of exposure to news and information about COVID-19 on social networks**					.02
	None	20.92 (9.46)			
	1	19.68 (9.53)			
	2 to 5	20.11 (9.41)			
	6 or more	20.91 (9.77)			
**Hours of exposure to news and information about COVID-19 on television**					.6
	None	20.05 (9.67)			
	1	20.07 (9.42)			
	2	20.50 (9.84)			
	3 or more	20.54 (9.38)			
**Hours of exposure to news and information about COVID-19 on the radio**					.60
	None	20.28 (9.53)			
	1 or more	20.46 (9.50)			

^a^One-way ANOVA between groups with Bonferroni correction and a significance level of *P*<.05.

**Table 4:** Association between infodemic variables and screening for geriatric anxiety (n=3307).

Variables		Presence of geriatric anxiety (participants), n (%)				*P* value^a^
		Yes		No		
**Frequency of exposure to social networks**						.008
	None (n=822)	143 (17.4)	679 (82.6)			
	Sometimes (n=1464)	194 (13.3)	1270 (86.7)			
	Often (n=1021)	173 (16.9)	848 (83.1)			
**Frequency of exposure to television**						.15
	None (n=394)	66 (16.8)	328 (83.2)			
	Sometimes (n=1440)	202 (14)	1238 (86)			
	Often (n=1473)	242 (16.4)	1231 (83.6)			
**Frequency of exposure to radio**						.13
	None (n=1956)	302 (15.4)	1654 (84.6)			
	Sometimes (n=956)	135 (14.1)	821 (85.9)			
	Often (n=395)	73 (18.5)	322 (81.5)			
**Hours of exposure to news and information about COVID-19 on social networks**						.01
	None (n=848)	145 (17.1)	703 (82.9)			
	1 (n=811)	112 (13.8)	699 (86.2)			
	2 to 5 (n=1084)	148 (13.7)	936 (86.3)			
	6 or more (n=560)	105 (18.8)	455 (81.2)			
**Hours of exposure to news and information about COVID-19 on television**						.62
	None (n=431)	69 (16)	362 (84)			
	1 (n=884)	127 (14.4)	757 (85.6)			
	2 (n=685)	102 (14.9)	583 (85.1)			
	3 or more (n=1301)	212 (16.3)	1089 (83.7)			
**Hours of exposure to news and information about COVID-19 on the radio**						.13
	None (n=2083)	306 (14.7)	1777 (85.3)			
	1 or more (n=1223)	204 (16.7)	1019 (83.3)			

^a^Chi-square test.

**Table 5:** Crude and adjusted logistic regression models for the Geriatric Anxiety Inventory score and crude and adjusted linear regression models for the Perceived Stress Scale score (n=3307).

Variables		Geriatric anxiety (logistic regression), OR^a^ (95% CI)			Perceived stress (linear regression), OR (95% CI)	
		Crude analysis	Adjusted analysis^b^	Crude analysis		Adjusted analysis^b^
**Frequency of exposure to social networks**						
	None	1	1	1		1
	Sometimes	0.72 (0.57 to 0.91)	0.69 (0.53 to 0.90)	–0.94 (–1.76 to –0.13)		–0.54 (–1.44 to 0.35)
	Often	0.96 (0.75 to 1.23)	0.92 (0.70 to 1.22)	–0.22 (–1.10 to 0.64)		0.25 (–0.73 to 1.23)
**Frequency of exposure to television**						
	None	1	1	1		1
	Sometimes	0.81 (0.59 to 1.09)	0.81 (0.59 to 1.10)	0.13 (–0.93 to 1.19)		0.04 (–1.02 to 1.10)
	Often	0.97 (0.72 to 1.31)	0.97 (0.72 to 1.31)	0.59 (–0.46 to 1.65)		0.49 (–0.57 to 1.56)
**Frequency of exposure to radio**						
	None	1	1	1		1
	Sometimes	0.90 (0.72 to 1.12)	0.88 (0.71 to 1.11)	0.03 (–0.69 to 0.77)		0.00 (–0.73 to 0.75)
	Often	1.24 (0.93 to 1.64)	1.24 (0.93 to 1.65)	–0.23 (–1.26 to 0.79)		–0.26 (–1.31 to 0.77)
**Hours of exposure to news and information about COVID-19 on social networks**						
	None	1	1	1		1
	1	0.77 (0.59 to 1.01)	0.73 (0.55 to 0.99)	–1.23 (–2.15 to –0.32)		0.89 (–1.88 to 0.11)
	2 to 5	0.76 (0.59 to 0.98)	0.74 (0.56 to 0.98)	–0.80 (–1.66 to 0.04)		–0.43 (–1.36 to 0.49)
	6 or more	1.11 (0.84 to 1.47)	1.10 (0.81 to 1.49)	–0.00 (–1.02 to 1.01)		0.43 (–0.65 to 1.53)
**Hours of exposure to news and information about COVID-19 on television**						
	None	1	1	1		1
	1	0.88 (0.64 to 1.21)	0.87 (0.63 to 1.20)	0.01 (–1.07 to 1.11)		–0.72 (–1.17 to 1.03)
	2	0.91 (0.65 to 1.28)	0.92 (0.65 to 1.28)	0.45 (–0.69 to 1.60)		0.37 (–0.78 to 1.52)
	3 or more	1.02 (0.75 to 1.37)	1.02 (0.75 to 1.39)	0.49 (–0.54 to 1.53)		0.33 (–0.72 to 1.39)
**Hours of exposure to news and information about COVID-19 on the radio**						
	None	1	1	1		1
	1 or more	1.16 (0.95 to 1.41)	1.16 (0.95 to 1.42)	0.17 (–0.49 to 0.85)		0.12 (–0.56 to 0.80)

^a^OR: odds ratio.

^b^Adjusted for gender, age group, education, cohabitation status, and income changes during the pandemic.

## Discussion

### Principal Findings and Comparison to Prior Work

This study found that older people, especially older women, were often exposed to information related to COVID-19 through television and social networks and that this had repercussions for their mental health, specifically GAD and stress.

The participants in this study were mostly White educated women aged 60 to 64 years who cohabited with 1 or 2 people in their own residence, received retirement income, and had no change in income due to the pandemic.

We hypothesize that our findings may be related to the income status of the study population, as this is a limitation for many older people. A lack of financial support and purchasing power limits access to the internet and to electronic devices. Social exclusion from internet access is considered one of the most important markers of socioeconomic inequality [9]. Moreover, the persistence of ageism can make older people digitally invisible, supporting the idea that older people do not have the necessary skills to make use of all the functions of technology [9,17].

One study inferred that older adults with 9 or more years of education are more likely to maintain internet use than older adults who had less education or who did not have access to school [18]. The proportion of internet users is higher among people with more education, those with a higher monthly family income, and those who are younger, which is in line with the results of this study. Older individuals with a lower educational level may have greater difficulty using and manipulating computers and cell phones and therefore use the internet less [17,18].

In line with this study, data from the Continuous Pesquisa Nacional por Amostra de Domicílios (National Household Sample Survey) [11] show that there are more women than men in Brazil. The Brazilian population is composed of 48.2% men and 51.8% women, and among older individuals, the percentage of women reaches 56%. In addition, women show greater receptivity, which ensures greater agreement and participation in opinion studies and interviews [19]. Media consumption increased during the period of social distancing, and although older people are part of the population with the lowest level of internet and digital media use, their access to and use of such media also increased during the pandemic [20,21].

Some studies indicate that social distancing and anxiety further increased digital screen time [20-23]. The use of the internet was promoted by video calls, which allowed greater contact between older people and their family and friends, and by apps that allowed delivery of food and medicine. These contacts, in addition to acting directly on health by promoting well-being and quality of life, helped reduce feelings of loneliness [22]. On the other hand, the continuous use of the internet and other means of communication contributed to increasing access to ill-organized information and continuous information overload [23].

This study found that television was the most used means of accessing information, followed by social networks. Older people reported that they were frequently exposed to information about COVID-19 on television and that their daily exposure was 3 hours or more.

Television is a medium that allows access to content produced by journalists, which suggests a certain level of confidence from the social point of view, although television may also promote content that is biased in favor of business, political, and even economic interests. During the pandemic, television briefly acquired centrality in Brazilian homes, because older people gathered around televisions in search of information about the disease. In emergency situations, social media is another useful channel for news, due to its rapid, real-time dissemination of specific, objective content [18,20].

Similarly, the literature shows that social networks and television are the main means of access to information in the pandemic [17,23-26]. A cross-sectional study conducted in Brazil with data from a virtual health survey revealed an increase in television-watching among adults during the pandemic [17].

A Brazilian study reported that during the pandemic television use averaged 3.31 hours, representing an increase of 1 hour and 45 minutes compared to the average time dedicated to television before the pandemic. Among the participants, those aged 60 years or older were the group who spent the longest average time watching television, which corroborates the data found in this study. The average time of use of computers or tablets was more than 5 hours during the pandemic, representing an average increase of 1 hour and 30 minutes compared to before the pandemic [27].

The hours and frequency of exposure to information are important data, because they can be indicative of information-seeking behavior, and they can potentiate infodemic content in the social context and the possible impacts of the infodemic on the mental health of the individual. Additionally, when investigating factors that increase protection and well-being regarding the use of the media, it is important to weigh the time spent in hours and the frequency of exposure to the media [6,28].

We found a significant association between infodemic variables, perceived stress, GAD, and the frequency of exposure to social networks. This suggests that exposure time and the source of information used by older people can impact their levels of anxiety and stress.

A recent German study reinforced the idea that the information conveyed by digital social media and traditional media, including broadcast television and radio, are linked to increased anxiety [28]. Conversely, the information provided by official sources and government websites is related to a decrease in anxiety [29]. Another study, conducted with 4872 adult Chinese citizens, found that older individuals who were frequently exposed to social media had a prevalence of anxiety of 22.6% and a prevalence of depressive disorders of 16.6% [14].

Research shows that in the context of the pandemic, being frequently exposed to alarming information about sick or deceased individuals increases the risk of mental disorders such as depression and anxiety [14,24,30]. Sensationalist content and misinformation generate a large supply of ideas that are difficult to process, causing symptoms of anguish and mental confusion.

In this study, mean perceived stress and GAD were higher among older participants who had no exposure to social networks. This agrees with studies [28-30] that point out that avoiding information can be an attempt at a defensive response and a form of emotional coping with infodemic stressors [28]. On the other hand, avoiding information can lead people to worry that they are missing important new information, generating stress and anxiety and showing that avoidance is a poor adaptive coping attitude [31].

The abovementioned German study showed that the choice to avoid information may be linked to a feeling that there is nothing that can be done to prevent the negative consequences of COVID-19. Participants who were “very concerned” about the risks to mental and physical health were also more likely to avoid or ignore the media. That analysis also revealed that a higher level of stress was associated with less access to social networks, especially among those who did not consider themselves to have good mental health [29].

Individuals tend to avoid exposing themselves to information when they associate it with aversive emotions or when the information might make it necessary to perform unwanted actions [31]. Indeed, the COVID-19 infodemic provoked thoughts about human mortality, sequelae of the disease, progression to the severe stage of the disease, and involved biosafety guidelines and recommendations that imposed new habits, including social distancing, to which a significant proportion of older people objected.

Thus, it is necessary to develop health education strategies appropriate to older individuals to respond to the infodemic and provide clear, specific, and objectively necessary information. In addition, it is important to investigate health literacy (ie, the ability to access and use information to make appropriate health decisions) and digital literacy (ie, the ability to locate information in digital media, select it, access it, and use it to acquire knowledge) [32,33].

The media constitute an important element in the construction of an informative discourse, distributing information about diseases and their methods of transmission, prevention, treatment, and immunization, as well as reporting on the most recent research [19]. Reliable and accessible information is important to increase the population’s awareness of preventive measures, the importance of immunization, and the fight against disease progression [19,32].

Given the above, we emphasize the importance of public health policies on infodemic management that encourage social education among older people on how to seek and interpret information. A past study that discussed the public health research agenda for managing infodemics suggested that public health authorities should develop, implement, validate, and adapt tools to manage infodemics at acute public health events, taking into account the country-specific context, and invest and foster policy makers in the scientific community [34].

### Limitations

A limitation of this study is the use of a web-based survey. This tool, which was chosen due to COVID-19 restrictions, limited the participation of many older people who do not have access to the internet or social networks, causing sample bias.

Another limitation was the profile of the participants, who were predominantly female, White, had completed elementary or higher education (even graduate education), lived in urban areas, cohabited with 1 or 2 people, had fixed incomes, and had incomes that were unaffected by the pandemic. These characteristics, which do not correspond exactly to the general Brazilian population, might have been protective against mental illness.

### Conclusions

Excessive information can affect mental health, cause feelings of stress and anxiety, and affect the quality of life of older individuals. In a pandemic context, the severity of these effects may be magnified by obstacles to sociable behavior and health service and support networks.

Investigation of the infodemic and its impacts on mental health should be part of the anamnesis of older people, so that they can share their feelings about this phenomenon and receive appropriate psychosocial care.

The management of excessive information should be a subject of debate in public health. The infodemic phenomenon is complex, because it is centralized, multifactorial, and cross-cut by important political and sociocultural issues.

This study contributes to the scientific community by presenting quantitative data that demonstrate the association between infodemic variables and mental health. This study also helps fill a knowledge gap in an important thematic axis and represents an original investigation into the impacts of the infodemic on GAD, perceived stress, and major depression in the older Brazilian population.

## Abbreviations

ANOVA: analysis of variance
GAD: generalized anxiety disorder
GAI: Geriatric Anxiety Inventory
PPS: Perceived Stress Scale

## Data Availability

The data sets generated and/or analyzed during this study are available from the corresponding author upon reasonable request.
